# Evaluation of Marginal Integrity of Four Bulk-Fill Dental Composite Materials: *In Vitro* Study

**DOI:** 10.1155/2015/701262

**Published:** 2015-03-22

**Authors:** Mirosław Orłowski, Bożena Tarczydło, Renata Chałas

**Affiliations:** Department of Conservative Dentistry and Endodontics, Medical University of Lublin, Ulica Karmelicka 7, 20-081 Lublin, Poland

## Abstract

*Objective*. The aim of the study was to compare under *in vitro* conditions marginal sealing of 4 different bulk-fill materials composite restorations of class II. *Methods*. Comparative evaluation concerned 4 composites of a bulk-fill type: SonicFill, Tetric EvoCeram Bulk Fill, Filtek Bulk Fill, and SDR. The study used 30 third molars without caries. In each tooth 4 cavities of class II were prepared. The prepared tooth samples were placed in a 1% methylene blue solution for 24 h, and after that in each restoration the depth of dye penetration along the side walls was evaluated. *Results*. The highest rating (score 0, no dye penetration) was achieved by 93.33% of the restorations made of the SDR material, 90% of restorations of SonicFill system, 86.66% of restorations of the composite Filtek Bulk Fill, and 73.33% of restorations of the Tetric EvoCeram Bulk Fill. *Conclusion*. The performed study showed that bulk-fill flowable or sonic-activated flowable composite restorations have better marginal sealing (lack of discoloration) in comparison with bulk-fill paste-like composite.

## 1. Introduction

The most essential factors determining preservation of restoration placed in a cavity are the marginal seal and absence of leakage [1, 2]. A marginal microleakage first defined by Kidd in 1976 is a process consisting in clinically undetectable penetration of bacteria, their metabolites, enzymes, toxins, ions, and other cariogenic factors between the filling and the cavity wall [3, 4]. Clinical consequences of microleakage are secondary caries, pulp inflammation, marginal discoloration, postoperative sensitivity, and the reduction of longevity of filling [5, 6]. It is believed that the existing occlusive load of the oral cavity and the thermal changes favor the formation of a marginal gap at the contact surface between the tooth and material [6, 7]. Rising expectations of patients regarding the aesthetics of fillings have recently made the composite resins the most commonly used nowadays restorative materials of lost tooth tissues. This applies to aesthetic dental restorations not only in the anterior teeth but also in the posterior teeth, so that in many countries composites do have almost totally replaced amalgam as restorative in posterior teeth [8]. Dentists expect from modern technology a composite material with high aesthetic value, less polymerization shrinkage, perfect marginal integrity, and relevant physicomechanical properties. If the material provides ease and short time of placement these are extremely desirable characteristics [7, 9, 10] but significant advances in composite technologies are not so frequent.

Embedding a composite restoration in posterior teeth is generally a time-consuming activity. The techniques of layers and thin 2 mm polymerization increments of the composites are widely recommended [11–13]. When extensive cavities are filled in posterior teeth, such a treatment can imply the risk of incorporating air bubbles or contaminants between the increments [14]. Manufacturers of composite materials, with a view to simplify the procedure of introducing the material into the cavity and its polymerization, now offer bulk-fill type composite resins. Simplification of procedures and shortening the time of embedding bulk-fill type restorations are due to possibility of applying a single up to 4 mm composite increment and it makes the work quicker by reducing the number of clinical steps. Thanks to high color translucency of these materials it is possible for the light to reach deeper but if the cavity is deeper than the maximum depth of cure 4 mm, it is necessary to apply another layer. The innovative system of polymerization initiation determines shortening of light-curing time and increasing the depth of cure. Low shrinkage of these materials and high filler content cause shrinkage stresses to be very low and this allows for application of thicker layers. The time of color matching process is shorter because of universal color of materials and shorter time of finishing and polishing of the restoration was noticed [6, 12]. Nevertheless an ideal bulk-fill composite would be one that could be placed into a preparation having a high C-factor design and still exhibited very little polymerization shrinkage stress, while maintaining a high degree of cure throughout [15].

The newly developed bulk-fill resins offer composites including low-viscosity (flowable) and high-viscosity (sculptable) material types. SDR (Smart Dentin Replacement) Posterior Bulk Fill Flowable Base is a single component, fluoride containing, and visibly light cured radiopaque resin composite restorative material. The composition is as follows: barium aluminofluoroborosilicate glass, strontium aluminofluorosilicate glass, modified urethane dimethacrylate resin, ethoxylated bisphenol A dimethacrylate (EBPADMA), triethylene glycol dimethacrylate (TEGDMA), camphorquinone photoinitiator, butylated hydroxytoluene (BHT), UV stabilizer, titanium dioxide, and iron oxide pigments. It has handling characteristics typical of a flowable composite but can be placed in 4 mm increments with minimal polymerization stress. SDR has a self-leveling feature that allows intimate adaptation to the prepared cavity walls. Available in one universal shade, it is designed to be overlaid with a methacrylate based universal/posterior composite for replacing missing occlusal/facial enamel. SonicFill system consists of a KaVo tip providing sonic application of a bulk-fill type composite by Kerr. Shrinkage stress compensation mechanism in SonicFill system was obtained using a resin having low shrinkage properties and high around 84% filler content. Other components are glass, oxide, chemicals (10–30%), 3-trimethoxysilylpropyl methacrylate (10–30%), silicon dioxide (5–10%), ethoxylated bisphenol A dimethacrylate (1–5%), bisphenol A bis(2-hydroxy-3-methacryloxypropyl) ether (1–5%), and triethylene glycol dimethacrylate (1–5%). Tetric EvoCeram Bulk Fill (Ivoclar Vivadent) is a nanohybrid composite with a monomer matrix containing dimethacrylates (20-21% weight). The fillers contain barium glass, ytterbium trifluoride, mixed oxide, and prepolymer (78%–81% by weight). Additional contents are additives, catalysts, stabilizers, and pigments (<1.0% weight). The total content of inorganic fillers is 76-77% weight or 53-54% volume. The particle size of the inorganic fillers is between 40 nm and 3,000 nm with a mean particle size of 550 nm. Tetric EvoCeram Bulk Fill contains in its composition an inhibitor of sensitivity to light and thus provides prolonged time for modeling of filling, an inhibitor of shrinkage stress in order to achieve optimal marginal seal, and Ivocerin, polymerization photoinitiator allowing curing of 4 mm layers of material. Filtek Bulk Fill (3M ESPE), a low-viscosity, visible-light activated flowable material for filling with bulk-fill technique, is manufactured in four shades (each of which may be polymerized in 4 mm increments according to international ISO standards) and two kinds of packaging, capsules and syringes. It contains Bis-GMA, UDMA, Bis-EMA, and Procrylat resins. Fillers are a combination of zirconia and silica having a particle size of 0.01–4.5 microns and ytterbium trifluoride filler having a particle size of 0.1–5.0 microns. The inorganic filler loading is approximately 64.5% by weight (42.5% by volume), Table 1.

A clinical evaluation of the new bulk-filling technique is important to observe the anatomical shape and marginal adaptation and margins discoloration. The occurrence of annual failure rates is also meaningful. Amongst many parameters defining the quality of materials that restore lost tooth tissues, marginal integrity seems to take part as the most important. During* in vitro* studies, various methods are used to detect the presence and assess the microleakage between the tooth tissues and filling material. Although a perfect marginal seal is not achievable clinically, a good marginal quality should be the main aim for clinicians. Marginal integrity has been evaluated using high magnification and penetrating dyes to reveal marginal gaps, both externally and internally [15]. The method is fast and easy to perform, which validates the choice of this method in studies [16]. The range of dye penetrations was assessed differently in millimeters or depending on cavity/tooth anatomy. The criteria are different and can be as follows: crossing dentin-enamel junction, width of the wall, width of the enamel/dentin layer, and number of walls penetrated by dye [17–19].

The aim of the study was to compare under* in vitro* conditions marginal sealing of composite restorations of class II cavities made of 4 different bulk-fill materials. The null hypothesis tested is that bulk-fill flowable composite resins do not lead to better marginal seal in comparison with bulk-fill paste-like composites.

## 2. Materials and Methods

### 2.1. Sample Preparations

In total 30 sound third molars, with neither carious lesions nor restorations, recently extracted for orthodontic reasons with the written agreement of every patient were selected for this* in vitro* study. After extraction, the teeth were cleaned from the remaining connective tissue and debris. Then, the teeth were rinsed with distilled water and stored at room temperature.

**Figure 1:**
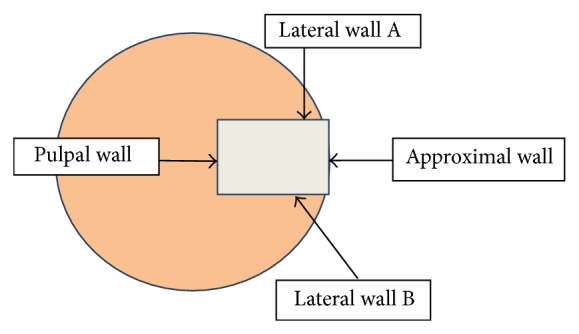
Graphic model of microleakage assessment on the transverse cross section.

Using a calibrated diamond bur under air-water cooling high speed handpiece, an experienced operator prepared in every tooth 4 cavities of class II to a depth of 4 mm (measured along the lateral wall), a width of 2 mm (pulpal wall), and length of 3 mm (approximal wall) (Figure 1). The margins of the cavities were finished with fine diamond bur.

### 2.2. Restorative Procedures

**Figure 2:**
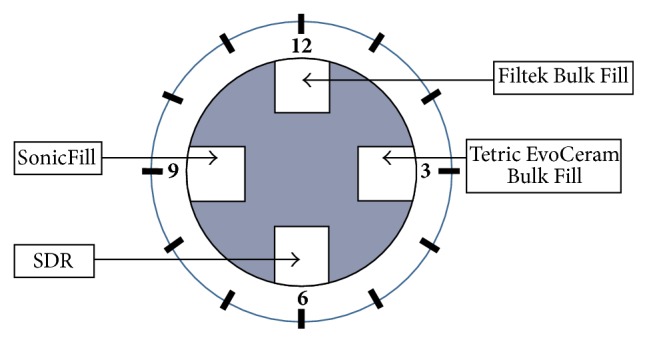
Order of restorations in every tooth sample.

For all samples the adhesive used was an etch-and-rinse system, applied following the manufacturer's instructions. All cavities were etched with total etch technique for 30 s, using 37% phosphoric acid, and rinsed with water. Then, the adhesive system was applied for all samples according to the restoration material used and polymerized. In every tooth 4 restorations of different bulk-fill materials were placed: SonicFill (Kerr and KaVo), Tetric EvoCeram Bulk Fill (Ivoclar Vivadent), Filtek Bulk Fill (3M ESPE), and SDR (Dentsply DeTrey). The application of all tested bulk-fill materials was performed in accordance with the manufacturer's instructions. SonicFill composite was inserted by sonic-activation using SonicFill handpiece, Filtek Bulk Fill, from a special syringe with the application dispenser, SDR from Compula tip using a dispensing device, and Tetric EvoCeram Bulk Fill with manual filling instruments and burnishers. Polymerization of materials took place with the use of LED lamps (Advanced TPCD, USA) spectrum 440–490 nm, power 900 mW/cm^2^. The restorations were finished with fine-grit diamond bur, mounted in a turbine with a water spray, and polished with graded abrasive discs and rubbers together with polishing paste (KerrHawe). Totally there were 120 restorations of 4 different types of composite bulk-fill materials placed. The order of restorations in every sample was always the same and based on a clockwise order: on 12 h, Filtek Bulk Fill, on 3 h, Tetric EvoCeram Bulk Fill, on 6 h, SDR, and on 9 h, SonicFill (Figure 2).

### 2.3. Microleakage Analysis

The teeth were dried and their tops were protected with pink wax and the smooth surfaces of the teeth (leaving a margin of 1 mm around the filling) were coated with nail varnish based on acetone (Inglot, Poland). The teeth were then placed for 24 hours in physiological saline to hydrate the teeth desiccated tissues. The prepared samples were placed in a 1% methylene blue solution for 24 h, after which the tooth surfaces were purified of the dye by means of rubbers and brushes with polishing compound.

For samples sections thus prepared teeth were cut with a diamond disc (0.5 mm Motyl, Poland) in the middle of the height of restorations parallel to the occlusal surface. In each restoration the depth of dye penetration was evaluated along the side walls. For the evaluation of dye penetration the Seliga optical microscope was used with a 10x magnification, pictures of the restoration interface were taken, and images were analyzed using a modified scale for bulk-fill materials with five-grade scale based on previous ones used in dental research studies [1, 7, 16–19]:

(0) no dye penetration into the filling material or along the filling-tooth interface,
(1) dye penetration into the filling material or along the filling-tooth interface up to half of the lateral wall A or B,
(2) dye penetration into the filling material or along the filling-tooth interface along all lateral wall A or B (till bottom of the cavity, pulpal wall),
(3) dye penetration into the filling material or along the filling-tooth interface up to half of both lateral walls A and B,
(4) dye penetration into the filling material or along the filling-tooth interface along both lateral walls A and B (till bottom of the cavity, pulpal wall).

The obtained results were statistically analyzed. Differences were considered statistically significant for *P* < 0.05.

## 3. Results

The condition of restorations made of bulk-fill composites expressed, as dye penetration, ranged from 0 till 4 and a detailed dye leakage analysis revealed differences in discoloration around the tested restorations.

**Figure 3:**
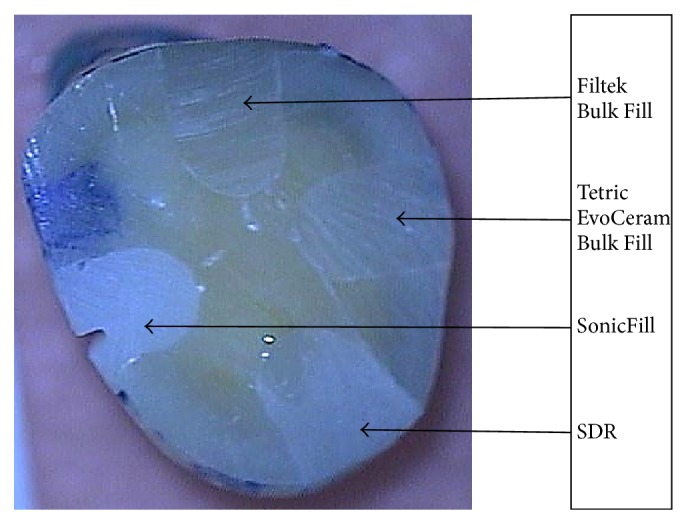
All restorations without microleakage (discoloration).

**Figure 4:**
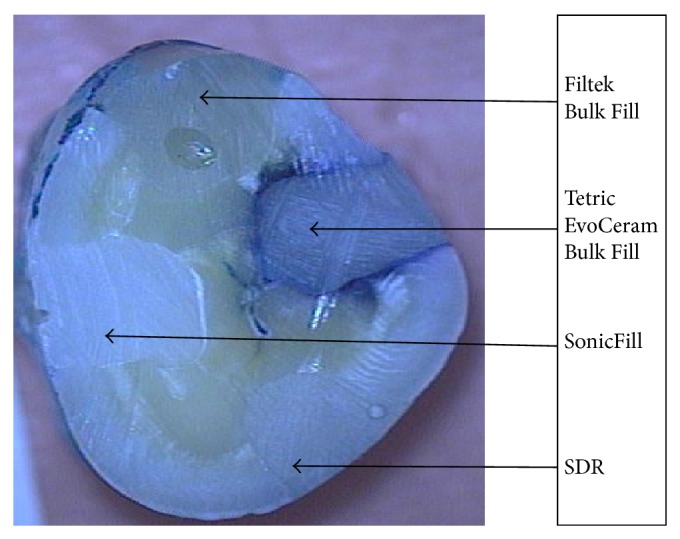
Discoloration of one of the restorations along the walls.

**Figure 5:**
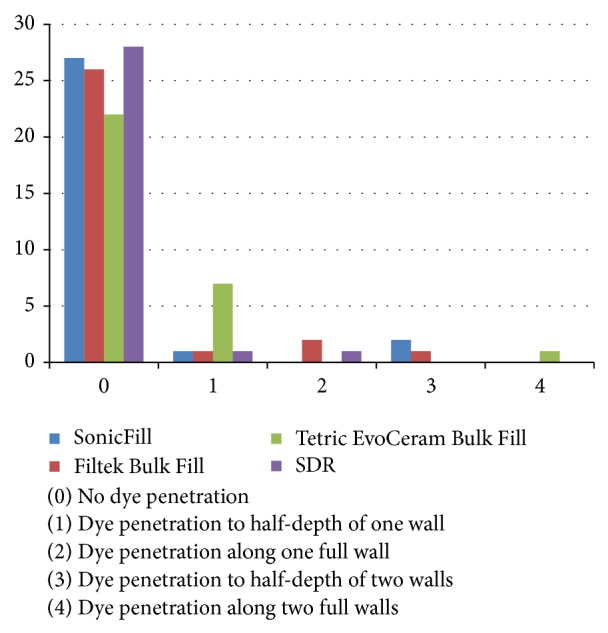
Dye penetration along examined walls.

Dye penetration rating using the grade scale was as follows (Table 2): the highest rating (score 0, no dye penetration) was achieved by 93.33% of the restorations made of the SDR material; 90% of restorations of SonicFill system; 86.66% of restorations of the composite Filtek Bulk Fill; and 73.33% of restorations of the Tetric EvoCeram Bulk Fill. The chosen tooth's sample without discoloration is presented in Figure 3. Score 1 (penetration of dye into half-depth of one wall) was achieved by 23.33% of restorations made of composite Tetric EvoCeram Bulk Fill and 3.33% of restorations from all other tested materials. Dye penetration along the entire length of one wall (score 2) was not found in the fillings made of the materials SonicFill and Tetric EvoCeram Bulk Fill and was observed in 6.66% of restorations made of Filtek Bulk Fill and 3.33% of the restorations made of SDR. The penetration of dye into half-depth of the two walls (score 3) of studied restorations was found only in the case of 6.66% of restorations of SonicFill and 3.33% of Filtek Bulk Fill restorations. Score 4 was achieved only by 3.33% restorations of the composite Tetric EvoCeram Bulk Fill. The chosen tooth's sample is presented in Figure 4. In these fillings, complete discoloration was seen on both walls. In the remaining materials tested, there was no discoloration of the two walls. All rates are graphically presented in Figure 5.

Due to the low percentage of negative samples and the test result being smaller than predetermined critical value, *χ* ^2^ test did not satisfy the condition of applicability. Thus, Fisher's exact test was used using a detailed comparison of the parameters. According to this test statistically significant differences were observed only between SDR and Tetric EvoCeram Bulk Fill restorations (*P* < 0.04), Table 3.

## 4. Discussion

Obtaining marginal integrity during filling cavities with composite materials determines tooth tissues protection against microleakage [1, 2, 20]. The biggest drawbacks of composite materials are polymerization shrinkage and thermal expansion greater than the expansion of the tooth. Polymerization shrinkage is responsible for the formation of internal stresses in the material and leakage between the filling and the walls of the cavity and the formation of posttreatment sensitivity [5, 6]. In order to reduce the risk of microleakage, the appropriate techniques should be applied that reduce the polymerization shrinkage [2, 12, 13, 20–23]. An important element in attempts to reduce the effects of the formation of internal stresses caused by polymerization shrinkage is an increase in the elasticity of the filler material and bonding system [6, 11, 20]. The increasingly common method of compensating stress is using a thin adhesive layer, the flowable composites [6, 24–26]. They have a lower modulus of elasticity so they are effective in reducing microleakage. It is generally believed that the conventional composite materials should be polymerized in increments not thicker than 2 mm [1, 27, 28]. During the polymerization of a thicker increment, the material can pass through the gel point at different times at different depths. When the superficial material layers are already in postgel phase, the deeper layers have not yet reached the gel point. The superficial part of the material becomes firm, and the deeper part is still liquid. Application of large increments of material triggers a shrinkage stress rise, and therefore the reduction of this phenomenon is a particular challenge. The recommended alternative to layered techniques, the bulk-fill techniques, has taken up this challenge. The single-increment application and polymerization method (the bulk-fill technique) proposed by the manufacturers of these composites did not compromise marginal adaptation of restorations. In assessing the integrity of the interphase tooth-filling, authors show differences resulting from the application technique [23, 27–29]. Abbas et al. and Federlin et al. obtained a lower degree of dye penetration in fillings made with layering technique than with one increment technique [17, 29]. The above quoted studies relate to restorations of the conventional composite materials. Bulk-fill composite materials evaluated in the present study seem to meet satisfactorily the requirements of this type of materials in terms of marginal adaptation. The dye penetration test showed no microleakage for high percentage (73.33–93.33%) of tested restorations. Bulk-fill composites are more translucent than other restorations, which allow the light to get to much deeper layers. The content of photoinitiators of polymerization and stress inhibitors determines the optimal marginal seal of these composites. The relationship between the method of filling cavities and marginal seal of composite fillings was also the subject of Skałecka-Sądel and Grzebieluch research [7, 20]. In the* in vitro* studies, they demonstrated that marginal integrity of class II fillings (preparation margin in enamel) of composite materials was higher when filling single increment of the material and lower with restorations of a layered material. Many factors affect the integrity of the bond between the tissues of the tooth and the material filling the prepared cavity. In addition to polymerization shrinkage, the C-factor, application method, and the polymerization of the composite resin play a significant role [1, 7, 20, 30–32]. In the present study the most favorable results were obtained when the application of the material took place using a SDR dispenser and activating sonic handpiece. It is in agreement with Ben-Amar et al. research conducted on the effect of the composite application and condensation on marginal seal [21]. Additionally, a higher marginal integrity and lower penetration of dye in fillings inserted using a sonic-activation condensing device were shown when compared with manual condensation. Statistically significant better marginal integrity of flowable tested materials, SDR, SonicFill, and Filtek Bulk Fill (compared to the composite Tetric EvoCeram Bulk Fill), may be due to their flow consistency during application. Peutzfeldt and Asmussen showed that the degree of fluidity when applying the composite material influences the marginal adaptation; increased fluidity of the composite makes it adhere better to the walls of the cavity [6]. The research by Ilie and Hickel implies that the flow composite materials based on SDR technology show a lower polymerization shrinkage compared with other flowable materials such as Filtek Supreme Flow and Esthet X Flow and also as compared to the nano- and microhybrid composites and based on silorans [33].

Report of Van Ende et al. seems to present interesting results of comparison studying three composites: conventional, liquid, and bulk-fill placed in the posterior teeth cavities of different cavity configurations coefficient (C-factor). The analyzed hypothesis was that the adaptation of the material to the cavity walls is not affected by C-factor, the type of the composite, and its application technique [31]. Verification of this hypothesis allowed the conclusion that the most satisfactory bonds with the tooth tissues were obtained when placing layered restorations in cavities with low C-factor, irrespective of the nature of the composite. On the other hand, in the cases where the C-factor was high, the choice of the composite proved important for the adaptation of the material [34]. Highly significant statistical difference was observed in the bond strength with tooth tissues between the flow-type composites and the conventional and the bulk-fill SDR resin. Markedly decreased bond strength was obtained in the case of flowable and conventional materials, in combination with a composite bulk-fill SDR material.

To interpret our results it should be also recognized that each bonding agent used, although specific for each material, may have influenced the marginal gap and this relationship between the bonding agent and the bulk-fill composite needs to be studied in the future. Additionally, bulk-fill materials may have different types of photoinitiators and thus require curing lights that activate them adequately [35]. That is why to avoid this kind of complication and to test these products as they are offered by manufacturer it was decided to study the bulk-fill materials together with a compatible bonding agent as integrated systems. It is also important to underline that the present results were obtained in the* in vitro* conditions such as a very good capability of light-curing device as well as direct access to the prepared tooth-composite samples. The achieved distance between the tip end of the light-curing device and the irradiated surface can hardly ever be obtained in working conditions in oral cavity of the patient where curing is less effective, which has been lately noticed [36].

## 5. Conclusions

Within the limits of this* in vitro* study, it can be concluded that bulk-fill flowable or sonic-activated flowable composite restorations have better marginal sealing in comparison with bulk-fill paste-like composites.

## Figures and Tables

**Table 1:** Bulk-fill composite materials used in the study.

Resin composite	Type	Manufacturer	Bonding agent	Maximum increment thickness recommended by manufacturer	Composition according to manufacturer's information	Number of restorations
Filtek Bulk Fill	Flowable	3M ESPE	Adper Single Bond 2	4 mm	Bis-GMA, UDMA, Bis-EMA, and Procrylat resins. Fillers are a combination of zirconia and silica having a particle size of 0.01–4.5 microns and ytterbium trifluoride filler having a particle size of 0.1–5.0 microns	30

SDR	Flowable	Dentsply DeTrey	XP Bond	4 mm	Barium aluminofluoroborosilicate glass, strontium aluminofluorosilicate glass, modified urethane dimethacrylate resin, ethoxylated bisphenol A dimethacrylate (EBPADMA), triethylene glycol dimethacrylate (TEGDMA), camphorquinone photoinitiator, butylated hydroxytoluene (BHT), UV stabilizer, titanium dioxide, and iron oxide pigments	30

SonicFill	Sonic flowable	Kerr	OptiBond Solo Plus	5 mm	Glass, oxide, chemicals (10–30%), 3-trimethoxysilylpropyl methacrylate (10–30%), silicon dioxide (5–10%), ethoxylated bisphenol A dimethacrylate (1–5%), bisphenol A bis(2-hydroxy-3-methacryloxypropyl) ether (1–5%), and triethylene glycol dimethacrylate (1–5%)	30

Tetric EvoCeram Bulk Fill	Packable	Ivoclar Vivadent	ExciTE F	4 mm	Monomer matrix containing dimethacrylates (20-21% weight). The fillers contain barium glass, ytterbium trifluoride, mixed oxide, and prepolymer (78%–81% by weight). Additional contents are additives, catalysts, stabilizers, and pigments (<1.0% weight)	30

**Table 2:** Dye leakage around examined restorations.

State of the restoration	SonicFill		Filtek Bulk Fill		Tetric EvoCeram Bulk Fill		SDR	
	*n*	%	*n*	%	*n*	%	*n*	%
No dye penetration	27	90	26	86,66	22	73,33	28	93,33
Dye penetration to half-depth of one wall	1	3,33	1	3,33	7	23,33	1	3,33
Dye penetration along one full wall	0	0	2	6,66	0	0	1	3,33
Dye penetration to half-depth of two walls	2	6,66	1	3,33	0	0	0	0
Dye penetration along two full walls	0	0	0	0	1	3,33	0	0
*N*/%	30	100	30	100	30	100	30	100

**Table 3:** Comparison of significant differences between pairs of composites.

	SonicFill	Filtek Bulk Fill	Tetric EvoCeram Bulk Fill	SDR
SonicFill	X	NS	NS	NS
Filtek Bulk Fill	NS	X	NS	NS
Tetric EvoCeram Bulk Fill	NS	NS	X	*P* < 0.04
SDR	NS	NS	*P* < 0.04	X
